# Temperament and Character Profiles of *Sasang* Typology in an Adult Clinical Sample

**DOI:** 10.1093/ecam/nep034

**Published:** 2011-02-20

**Authors:** Soo Hyun Park, Myoung-geun Kim, Soo Jin Lee, Jong Yeol Kim, Han Chae

**Affiliations:** ^1^Department of Occupational Therapy, College of Health Sciences, Yonsei University, Wonju, Republic of Korea; ^2^Korea Institute of Oriental Medicine, Daejeon, Republic of Korea; ^3^Department of Psychology, Yonsei University, Seoul, Republic of Korea; ^4^Division of Longevity and Biofunctional Medicine, School of Korean Medicine, Pusan National University, Yangsan, Kyungnam, 626-770, Republic of Korea

## Abstract

The purpose of this study was to examine the biopsychological personality profiles of traditional Korean *Sasang* typology based on the Temperament and Character Inventory (TCI) in a Korean adult clinical sample. A total of 97 adults completed the Korean version of the TCI. The participants were classified as one of three traditional Korean *Sasang* types (31 So-Yang, 41 Tae-Eum, 25 So-Eum) by three specialists in *Sasang* typology. The seven dimensions of TCI were compared between the different *Sasang* types using analysis of variance (ANOVA) and profile analysis. There were no significant differences in age, gender and education across the *Sasang* types. The TCI profile for each of the *Sasang* types was significantly different (profile analysis, df = 5.038, *F* = 3.546, *P* = .004). There were significant differences in the temperament dimensions of Novelty Seeking (*F* = 3.43, *P* = .036) and Harm Avoidance (*F* = 5.43, *P* = .006) among the *Sasang* types. The Novelty Seeking score of the So-Yang type (31.90 ± 9.87) was higher than that of the So-Eum type (25.24 ± 9.21; *P* = .019) while the So-Eum type (44.64 ± 8.47) scored higher on the Harm Avoidance score compared to the So-Yang type (35.16 ± 11.50; *P* = .003). There were no significant differences in the temperament dimension of Reward Dependence and Persistence, and the three character dimensions of Self-Directedness, Cooperativeness and Self-Transcendence. Results demonstrated distinct temperament traits associated with traditional Korean *Sasang* types using an objective biopsychological personality inventory. With further study, the *Sasang* typology may lead to enhanced clinical safety and efficacy as part of personalized medicine with traditional medicine.

## 1. Introduction

Current changes in medicine have led researchers to focus on preventive, predictive and personalized medicine. Personalized medicine represents a form of individualized medicine wherein medical treatment is tailored to the individual to maximize efficacy and safety. A diverse array of research is being conducted utilizing genetic characteristics following the Human Genome Project [1]. *Sasang* typology, as a traditional Korean medical typology, epitomizes personalized medicine based on both physical and psychological characteristics and features, and shares much similarity with the Western academic tradition [2–9]. For instance, just as Kretschmer [10] and Sheldon [11] identified different human personality types based on body type and psychological characteristics, *Sasang* typology not only encompasses such a similar constitutional classification system, but also shares a similar conceptual basis with Eysenck's description of introversion-extroversion and neuroticism [2, 3, 12]. In particular, *Sasang* typology also appears to be related to Hippocrates' concept of the four humors [3, 13, 14].

*Sasang* typology, originally theorized by Lee Je-ma [15] is widely used in the clinical diagnosis and treatment of disease in Korea. In *Sasang* typology, one's physical constitution is classified according to the traits of an individual's mind and body, with the implication that one's sensitivity and response to certain drugs can be different depending on one's *Sasang* type [2]. There are four *Sasang* types: Tae-Eum, So-Eum, So-Yang and Tae-Yang. The So-Yang type, for instance, is a sharp and clean-looking person who is extroverted, hot-tempered and interested in the outside world. The So-Eum type is an inactive, prudent, narrow-minded, negative, organized, nervous and resolute person.

Research focusing on the psychological and personality characteristics of different *Sasang* types has revealed a variety of psychological profiles [3]. For example, the So-Yang types are extroverted, while the So-Eum types scored higher on the judging versus perceiving dimension of the Myers Briggs Type Indicator (MBTI) compared to the So-Yang and Tae-Eum types. Furthermore, in studies by Chae and colleagues [2, 3], while the So-Yang and So-Eum types share similar physical characteristics, there exist significant psychological differences. Through systematic review of research pertaining to *Sasang* typology, neuroticism and extroversion were determined to be important personality factors in discriminating between So-Yang and So-Eum types [3]. More specifically, the So-Yang type demonstrated high extraversion and low neuroticism, while the So-Eum type exhibited low extraversion and high neuroticism. Interestingly, these two factors have been repeatedly mentioned as perhaps the most critical variables in a variety of personality research, such as Eysenck's theory of personality [12] and Costa and McCrae's five-factor theory of personality [16, 17]. The descriptive characteristics of personality outlined by Eysenck, namely the four personality types of Stable-Extrovert, Neurotic-Extrovert, Stable-Introvert, and Neurotic-Introvert, share remarkable similarity with Galen's Sanguine, Choleric, Phlegmatic, and Melancholic types and the Tae-Yang, So-Yang, Tae-Eum and So-Eum *Sasang* types [3, 13, 14].

Activity versus inactivity as personified by the So-Yang and So-Eum types share similarities with Gray's personality theory. In other words, Gray's personality theory is based on the principle that individual differences in personality reflect the variation in sensitivity and responsiveness to punishment and frustrative non-reward, and reward and relieving non-punishment, the behavioral inhibition system (BIS) and the behavioral activation system (BAS), respectively [18, 19]. Chae and colleagues had previously implicated the BAS and BIS system as the foundation for medical and clinical use of personality traits in traditional Korean *Sasang* typology [3]. All of these results imply the importance of clinical research on *Sasang* typology from a biopsychological standpoint.

The Temperament and Character Inventory (TCI) originally developed by Cloninger, is an assessment tool based on such a biopsychological personality model that may reflect the BAS/BIS concept [19–21]. This possibility is based on the fact that Cloninger's HA dimension is markedly similar to Gray's Anxiety dimension when looking at the pattern of factor loadings. However, Cloninger's NS dimension is reportedly not compatible with Gray's Impulsivity dimension [22]. The TCI consists of four temperament dimensions and three character dimensions. Novelty Seeking (NS) is viewed as a heritable bias in the activation or initiation of behaviors such as frequent exploratory activity in response to novelty, impulsive decision-making, extravagance in approach to cues of reward, and quick loss of temper and active avoidance of frustration. Harm Avoidance (HA) is viewed as a heritable bias in the inhibition or cessation of behaviors, such as pessimistic worry in anticipation of future problems, passive avoidant behaviors such as fear of uncertainty and shyness of strangers and rapid fatigability. Reward Dependence (RD) is viewed as a heritable bias in the maintenance or continuation of ongoing behaviors and is manifested as sentimentality, social attachment and dependence on approval of others. Persistence (P), which ultimately separated from the Reward Dependence temperament factor as an independent fourth temperament dimension, is viewed as a heritable bias in terms of perseverance despite frustration and fatigue. Differences between individuals based on these temperament dimensions can influence the outcome of medical treatment [23], and are observable from early childhood and moderately predictive of adolescent and adult behavior. Consequently, such temperament factors appear to be heritable, manifest early in life and apparently involve pre-conceptual and unconscious biases in learning.

In contrast, character factors appear to mature in adulthood and affect personal and social effectiveness through insight learning about self-concepts. Character dimensions that are based on theories of social, cognitive and personality development are Self-Directedness (SD), Cooperativeness (C) and Self-Transcendence (ST), each referring to individual differences in self-concepts, goals and values. In contrast to temperament, aspects of character are thought to be influenced by social learning along with genetic factors and to mature in stages throughout the lifespan [20, 24, 25]. Self-Directedness implies an autonomous self-concept and feelings of hope, honor and self-confidence. Cooperativeness involves identification with and acceptance of others, compassion and charity. Finally, Self-Transcendence is related to spirituality, patience and self-forgetfulness.

Here, we examined the temperament and character profiles measured by the TCI based on *Sasang* typology in a clinical sample of community-dwelling adult men and women. Our research sought to corroborate results of an earlier study [2] that found a clear difference in the biopsychological characteristics of the So-Yang versus So-Eum types. Secondly, only individuals whose *Sasang* types were confirmed by three clinical specialists are included. Earlier research distinguished between *Sasang* types by using a self-report questionnaire, thereby making direct comparison with reactivity to medication difficult [2]. By using a clinical sample that had undergone *Sasang*-based medical regimen, we hope that a guideline for use of herbal medication will be provided for future reference.

## 2. Materials and Methods

This research is part of the Lee Je-Ma Project, a national research study investigating the characteristics of *Sasang* types with the goal of providing a scientific and empirical basis of *Sasang* medicine. Our approach passed the IRB of the Korea Institute of Oriental Medicine, and participants completed a written consent form.

### 2.1. Subjects

**Figure 1:**
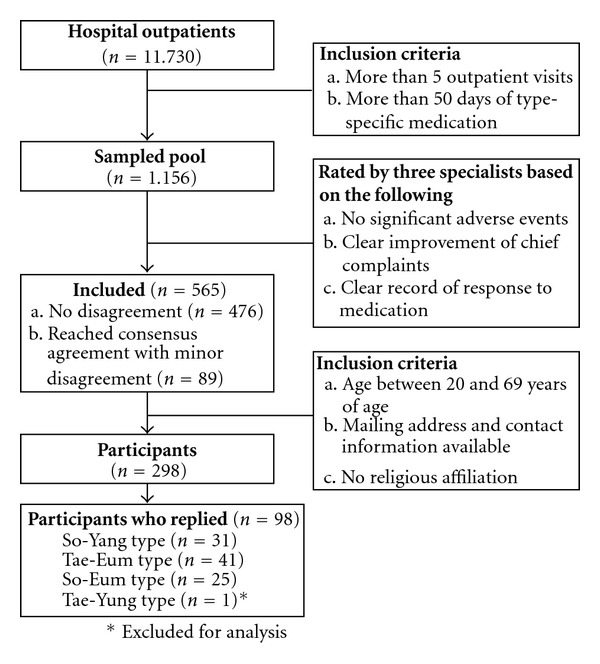
Flow diagram for the detailed procedure of participant selection.

Participants were selected in 2008 from a pool of 11 730 outpatients who visited a hospital for Korean Oriental Medicine between June of 1996 and May of 2004 in several major cities of Korea such as Kwangju, Jeonju and Seoul. Among these participants, 1156 individuals who met all of the following inclusion criteria composed the initial sample pool: (i) made a minimum of five outpatient visits, (ii) underwent pharmacological management prescribed for the particular *Sasang* type for 50 days or more, (iii) did not manifest significant medication adverse events as indicated in the medical records, (iv) demonstrated clear improvement in their chief complaints and pre-existing symptoms and/or exhibited a unique improvement pattern and (v) for whom there existed clear documentation of medication treatment response in the medical chart (Figure 1). Clinical determination of one's *Sasang* typology was based on the medical chart review conducted by three licensed medical specialists in Korean *Sasang* typology who have been in clinical practice for 5 years or more. The confidence level of each individual's *Sasang* type classification was rated as either low, medium and high, and only 565 patients for whom the confidence level was high were selected. Confidence level was determined to be high when no disagreement in *Sasang* typology classification among the three specialists was found or when a minor disagreement arose but which resulted in consensus agreement following discussion among the raters. Among the 565 individuals, those (i) who were younger than 20 years of age or older than 69 years of age, (ii) who did not provide a mailing address and/or contact information or (iii) individuals with religious affiliations were excluded from the study. A total of 298 individuals were mailed the TCI, and 97 individuals returned the questionnaire in 2008. The prevalence of the Tae-Yang type is historically found to be extremely low (0.03–0.1%) [15, 26] and only one individual (65-year-old female) was classified as a Tae-Yang type in the present study. As such, only data pertaining to the Tae-Eum (*n* = 41), So-Eum (*n* = 25) and So-Yang (*n* = 31) *Sasang* types were statistically analysed.

### 2.2. Measure

The Korean version of the Temperament and Character Inventory-Revised-Short (TCI-RS) [21] is a 140-item self-report questionnaire that asks individuals to rate each item on a 5-point scale (0 = *not at all* to 4 = *very true*). The Korean version of the questionnaire was standardized and validated in 2007 and demonstrated validity and reliability. Each of the dimensions of the TCI is assessed as the sum of scores on three to five subscales measuring 29 more specific traits that define the temperament and character dimensions. For example, Harm Avoidance includes anticipatory worry and pessimism versus uninhibited optimism (HA1), fear of uncertainty (HA2), shyness with stranger (HA3) and fatigability versus vigor (HA4). Overall internal consistency as measured by Cronbach's alpha was 0.871. Cronbach's alpha for the NS, HA, RD, P, SD, CO and ST scales were 0.829, 0.857, 0.814, 0.821, 0.865, 0.758, and 0.899.

### 2.3. Statistical Analysis

Demographic differences between *Sasang* types (*So-Yang*, *Tae-Eum* and *So-Eum*) were tested using Analysis of Variance (ANOVA) for continuous variable (age) and Fisher's exact tests for categorical variables (level of education, gender). Profile analysis such as test of parallelism and flatness was performed to test the difference of the TCI profile for each Sasang type. Analyses of Variance (ANOVAs) were conducted to test between-group differences in TCI scores across the three *Sasang* types. The data are represented as means and standard deviations. All analyses were conducted using SPSS 16.0.1 for Windows (SPSS Inc., Chicago, IL).

## 3. Results

Results are divided into two sections. The first describes the background characteristics of the sample, and the second describes the TCI temperament and character profiles across the three *Sasang* types.

### 3.1. Demographic Background

The mean age, gender composition and educational attainment level are described in Table 1.

The mean age of the entire sample was 47.6 ± 10.5 years. There were no significant differences in age, gender composition and level of education between the three *Sasang* types, *F*(2,94) = 1.94, *P* = .149, *χ* ^2^ = 0.871, *P* = .647 and *χ* ^2^ = 0.764, *P* = .682, respectively.

### 3.2. TCI Profile across the *Sasang* Types

**Figure 2:**
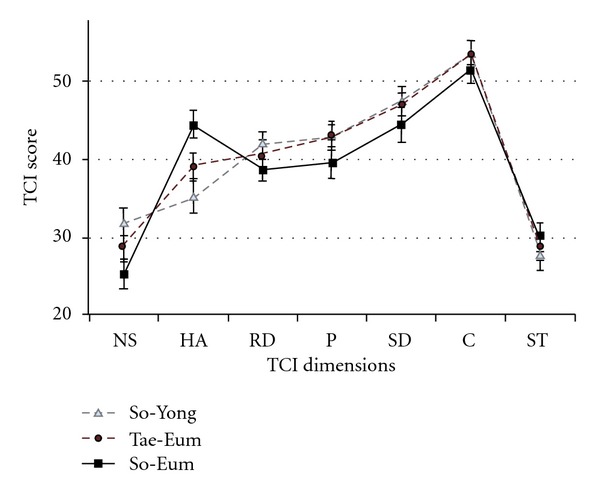
TCI dimension score profile of *Sasan*g types. The TCI score profile of the So-Yang, Tae-Eum and So-Eum types were significantly different (flatness with Greenhouse-Geisser test, df = 2.519, *F* = 33.4, *P* < .001; parallelism with Greenhouse-Geisser correction, df = 5.038, *F* = 3.546, *P* = .004). The So-Yang types (31.90 ± 9.87) scored higher on the NS scale than the So-Eum types (25.24 ± 9.21), *F*(2,94) = 3.43, *P* = .04. The So-Eum types (44.64 ± 8.47) scored higher on the HA scale than the So-Yang types (35.16 ± 11.50), *F*(2,94) = 5.43, *P* = .01. Whisker represents standard errors. NS: novelty-seeking; HA: harm-avoidance; RD: reward-dependence; P: persistence; SD: self-directedness; C: cooperation; ST: self-transcendence.

Figure 2 demonstrates the TCI temperament and character profile for each of the three *Sasang* types. The TCI temperament score profile of the So-Yang, Tae-Eum and So-Eum types were significantly different. The profile of TCI temperament dimension, such as NS, HA, RD and *P* was not flat (Greenhouse-Geisser test, df = 2.519, *F* = 33.4, *P* < .001). As for the parallelism of TCI temperament profile with the interaction of *Sasang* type were significantly different (Greenhouse-Geisser test, df = 5.038, *F* = 3.546, *P* = .004). *Sasang* types showed significant differences in the Novelty Seeking, *F*(2,94) = 3.43, *P* = .036, and in the Harm Avoidance, *F*(2,94) = 5.43, *P* = .006, scales of the TCI. Dunnett's post-hoc tests revealed that the So-Yang type (31.90 ± 9.87) scored higher on the NS scales compared to the So-Eum type (25.24 ± 9.21; *P* = .019) and lower on the HA scale (35.16 ± 11.50), compared to the So-Eum type (44.64 ± 8.47; *P* = .003). There were no other significant differences between *Sasang* types in the other 5 TCI dimensions.

**Figure 3:**
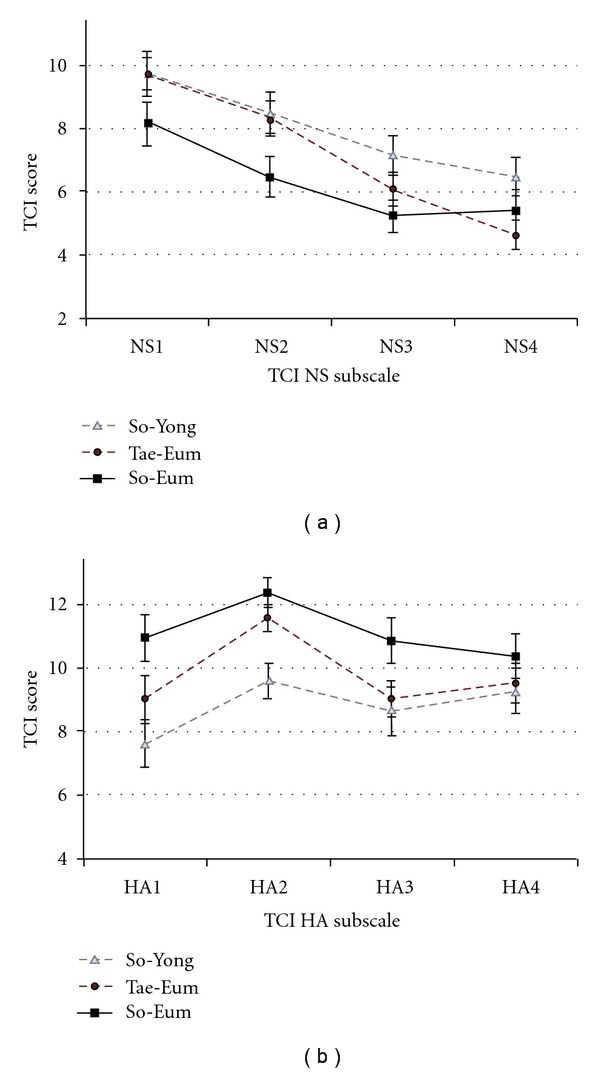
(a) TCI NS subscale scores of *Sasang* types. There were no significant differences between *Sasang* types. NS1: Exploratory Excitability versus Stoic Rigidity; NS2: Impulsiveness versus Reflection; NS3: Extravagance versus Reserve; NS4: Disorderliness versus Regimentation (b) TCI HA subscale scores of *Sasang* types. The So-Eum types scored higher on the HA1 (10.96 ± 3.69) and HA2 (12.40 ± 2.36) subscales than the So-Yang types (7.61 ± 4.26 and 9.61 ± 3.14), *F*(2,94) = 4.06, *P* = .02 and *F*(2,94) = 7.59, *P* = .001, respectively. Whisker represents standard errors. HA1: anticipatory worry and pessimism versus uninhibited optimism; HA2: fear of uncertainty; HA3: shyness with stranger; HA4: fatigability versus vigor.

To further explore these dimensions, subscales of the NS and HA scales were analysed (Figure 3). A significant difference was found in the HA1 (Anticipatory Worry) and HA2 (Fear of Uncertainty) subscales, *F*(2,94) = 4.06, *P* = .02 and *F*(2,94) = 7.59, *P* = .001. More specifically, the So-Eum type scored higher on the HA1 (10.96 ± 3.69) and HA2 (12.40 ± 2.36) subscales when compared to the So-Yang type (7.61 ± 4.26 and 9.61 ± 3.14). Such differences make intuitive sense, based on the traits attributed to each *Sasang* type. In addition, the difference between *Sasang* types on the NS4 (Disorderliness) subscale approached statistical significance, *F*(2,94) = 2.98, *P* = .056, with So-Yang type (6.48 ± 3.39) scoring higher compared to the Tae-Eum type (4.63 ± 2.95).

### 4. Discussion

In the current study, the relationship between objective personality theory as described by Cloninger in his biosocial model [27] of temperament and character and *Sasang* typology of traditional Korean medicine was examined. According to our hypothesis, distinct temperament and character profiles would emerge for the different *Sasang* types as indicated on the TCI [3].

The results demonstrated that the TCI temperament dimensions of Novelty Seeking and Harm Avoidance were particularly important in distinguishing between the three *Sasang* types, especially between the So-Eum and the So-Yang types. The So-Yang type, exhibiting contrasting features from the So-Eum type, scored higher on the Novelty Seeking scale and lower on the Harm Avoidance scale of the TCI. As based on such a TCI profile, they can be described as quick-tempered, excitable, impulsive and vigorous [19]. The So-Yang type was originally described as hot-tempered, easily bored and righteous by Lee Je-ma [15]. Such results also corroborate findings from previous research that used a different diagnostic tool to distinguish between the *Sasang* types [3].

Looking more closely at the subscales of the NS and HA dimensions, the fact that the So-Eum type scored significantly higher on the HA1 (Anticipatory worry and pessimism versus Uninhibited optimism) and HA2 (Fear of Uncertainty) compared to the So-Yang type portrays them as pessimistic worriers who tend to anticipate harm and failure and experience difficulty tolerating uncertainty. Such temperament descriptions also coincide with Lee Je-ma's original theory that describes the So-Eum type as negative, nervous and mild. Such corroborated empirical findings support the argument that *Sasang* typology, as based in mind-body medicine, can serve as a scientific basis for providing personalized and integrative medicine, and the importance of considering both physical traits and psychological characteristics in tailoring medical interventions.

The *Sasang* typology encompasses prescription guidelines for type-specific medical herbs for optimal efficacy and safety based on one's *Sasang* type that has accumulated through the 5000-year history of clinical experiences [15, 28]. Key representative herbs based on *Sasang* typology are provided in Table 2. The medical herbs listed in the table are used in 50–60% of all type-specific prescriptions (mixture of medical herbs) and herbs used for one particular *Sasang* type are not used for another *Sasang* type. For individuals who are low in NS and high in HA (i.e., So-Eum type), ginseng may be quite effective. However, clinicians may consider adverse treatment effects of ginseng for individuals who are high in NS and low in HA (i.e., So-Yang type).

An exploratory question revolved around whether temperament factors, compared to character factors, will contribute more to predicting the stability of *Sasang* typology. As in previous research, differences were found in the NS and HA dimensions, two temperament dimensions which are presumed to have a genetic and anatomical basis and can influence the clinical outcomes [17, 19, 23, 29–32]. However, no significant differences were found between the *Sasang* types in the character dimensions, which are presumed to be able to change over time. This lends support, albeit indirectly, for the argument that the *Sasang* typology demonstrates longitudinal stability in terms of temperament and can be considered as biopsychological in nature. More specifically, NS and HA appear to be theoretically related to Gray's BAS and BIS [19], respectively, and this study lends partial support for the relationship between the So-Yang type, Novelty Seeking and BAS, and between the So-Eum type, Harm Avoidance and BIS.

Several limitations of this study should be acknowledged. First, individuals who chose not to participate by not returning the survey packet may represent a different slice of the population than those represented in this particular sample, suggesting the possibility of representational bias. However, given that the prevalence of the Tae-Eum type is approximately 50% and that of the So-Yang and So-Eum types are approximately 25% each [15, 26], the sample ratio appears to be consistent with extant research.

Second, we did not incorporate multi-method means of classifying *Sasang* typology. Although only those participants who were rated by three experienced specialists in *Sasang* typology as a particular *Sasang* type at a high confidence level based on response to medication, future studies should utilize a diverse source of *Sasang* type classification to enhance reliability such as the combination scores from the research- and clinical-oriented methods.

Third, although significant differences between the So-Yang and So-Eum types were found in the *post-hoc* analyses, a significant difference was not found with the Tae-Eum type. However, a unique difference was observed between the Tae-Eum versus the So-Yang and So-Eum types when physical characteristics were examined [2, 3]. It is unclear whether such findings are due to our small sample size or due to the possibility that the Tae-Eum type represents a middle ground between the psychological characteristics of the So-Yang versus the So-Eum types. The systematic review results by Chae et al. [3] suggest that the current trend cannot simply be explained by the small sample size but may indicate that the psychological characteristics of the Tae-Eum type lie somewhere in between the So-Yang and So-Eum types. Future studies should utilize a larger sample size and various age cohorts to empirically ascertain the psychological characteristics of the Tae-Eum type.

Lastly, the nature of the So-Yang type is characterized by the feature of anger, a component of negative affect [33], and the nature of the So-Eum type is characterized by the feature of enjoyment, a component of positive affect [34] according to the *Dongyisoosebowon* [15] and *Yellow Emperor*'*s Classic of Internal Medicine* [35]. At first glance, it would appear that the affect of the So-Yang and So-Eum types would be characterized as the exact opposite. However, it is also possible that there exist certain mediating and/or controlling factors that may influence affectivity itself and/or its outward expression [5, 6, 31]. Follow-up studies are needed to examine what types of affectivity in Western academic tradition the So-Yang versus the So-Eum types would exhibit.

In summary, two points should be emphasized. First, our analysis revealed stable biopsychological differences between the So-Yang and So-Eum *Sasang* types and indicates a need for future studies that examine more closely at the neuroscience of the BAS/BIS system as well as the positive and negative affect related to *Sasang* types. Second, utilization of our results may lead to more enhanced clinical safety and efficacy as part of personalized medicine in medication use and may provide an important clinical guideline with individual's temperament in the use of medical herbs.

### Funding

This work was supported by the Korea Science and Engineering Foundation (KOSEF) grant funded by the Korea government (MEST) (No. M10643020004-08N4302-00400).

## Figures and Tables

**Table 1:** Demographic characteristics across *Sasang* types.

Demographic variables	So-Yang (*n* = 31)	Tae-Eum (*n* = 41)	So-Eum (*n* = 25)
Age (mean, SD)	44.6 (11.2)	49.1 (10.7)	48.8 (8.5)

Gender			
Male (%)	11 (35.5)	19 (46.3)	10 (40.0)
Female (%)	20 (64.5)	22 (53.7)	15 (60.0)

Level of education			
Middle school (%)	1 (3.2)	6 (14.6)	3 (12.0)
High school (%)	6 (19.4)	3 (7.3)	7 (28.0)
College (%)	12 (38.7)	15 (36.6)	6 (24.0)
Master Degree (%)	6 (19.4)	7 (17.1)	7 (28.0)
Doctorate Degree (%)	3 (9.7)	4 (9.8)	1 (4.0)

Results are reported as means (standard deviations) or as numbers (%). No significant differences in frequency of age, gender and education between types.

**Table 2:** Representative type-specific medical herbs for each *Sasang* type.

*Sasang* type	Medical herbs
Tae-Yang	Chaenomelis Fructus, Acanthopanacis Cortex, Phragmitis Rhizoma
So-Yang	Rehmanniae Radix, Corni Fructus, Poria, Alismatis Rhizoma, Ostericii Radix, Angelicae Pubescentis Radix
Tae-Eum	Ephedrae Herba, Liriopis Tuber, Schisandrae Fructus, Dioscoreae Rhizoma, Platycodi Radix, Coicis Semen, Puerariae Radix
So-Eum	Ginseng Radix, Atractylodis Rhizoma White, Glycyrrhizae Radix, Cinnamomi Cortex, Citri Pericarpium, Zingiberis Rhizoma Crudus
